# Population preference values for health states in relapsed or refractory B-precursor acute lymphoblastic leukemia in the United Kingdom

**DOI:** 10.1186/s12955-015-0377-3

**Published:** 2015-11-16

**Authors:** Mike Aristides, Arie Barlev, Beth Barber, Merel Gijsen, Casey Quinn

**Affiliations:** PRMA Consulting, Fleet, UK; Amgen GHE, Thousand Oaks, USA

**Keywords:** Leukemia, Lymphoblastic, Acute, Precursor B-cell, Health-related quality of life, Stated preference, Utility value

## Abstract

**Background:**

To date, reliable and comprehensive health-related quality of life data for patients with relapsed or refractory B-precursor acute lymphoblastic leukemia (ALL) have not been collected in clinical trials of the disease, and no utility studies have been published. The purpose of this study was to define and validate health states experienced by adults with relapsed/refractory B-precursor ALL, and to assign utility values to these health states using time-trade off methodology.

**Methods:**

This study was conducted in the UK in three phases. In the first phase, five health state descriptions were developed based on a recent clinical trial. The second phase validated the health state descriptions with clinicians and patients with experience of relapsed/refractory B-precursor ALL. The third phase involved prospective health state valuation using time-trade off methodology in a sample of the general public. The study was approved by the UK National Health Service Research Ethics Committee.

**Results:**

In total, 123 participants were recruited and included in the final analysis; all participants gave written, informed consent. Complete remission was the most preferred health state (mean utility [SEM], 0.86 [0.01]), followed by complete remission with partial hematological recovery (with minimal risk of bleeding or developing infection) (0.75 [0.02]); aplastic bone marrow (0.59 [0.02]); partial remission (0.50 [0.03]); and progressive disease (0.30 [0.04]).

**Conclusions:**

This is the first study to report utility values for health states associated with relapsed/refractory B-precursor ALL. It was designed and conducted to align with NICE guidance on alternative methods for generating health state utility values when EQ-5D data are either unavailable or inappropriate. These utilities can be applied in future cost-effectiveness analyses of treatment for relapsed/refractory B-precursor ALL.

**Electronic supplementary material:**

The online version of this article (doi:10.1186/s12955-015-0377-3) contains supplementary material, which is available to authorized users.

## Background

Acute lymphoblastic leukemia (ALL) is a rare and aggressive disease; in 2011, there were approximately 650 new cases of ALL in the UK, representing only 0.2 % of all new cancer cases [1]. Although first-line treatments for B-precursor ALL have improved survival rates, a considerable proportion of patients are refractory to treatment or experience relapse [2–5]. Adults with relapsed or refractory B-precursor ALL have a very poor prognosis; the main aim of treatment is to achieve a durable remission without compromising health-related quality of life (HRQL) [2–5]. However, the HRQL of adults with relapsed or refractory B-precursor ALL, including the impact of their treatment, is not well understood. This is likely due to the rarity of the disease.

In many countries, the decision to approve novel therapies is informed by calculation of the cost per quality adjusted life-year, which requires utility data to quantitatively compare health states alongside survival [6, 7]. Generally, HTA agencies prefer utility data derived directly from patients in a clinical trial, using the EQ-5D [6–8]. The EQ-5D is a standardized measure of health status, and is applicable to a wide range of health conditions and treatments [9]. To date, neither EQ-5D nor other HRQL data have been collected in clinical trials of B-precursor ALL.

When EQ-5D data are unavailable, alternative methodologies for generating utility values, such as the use of preference elicitation, can be used. Preference elicitation may also be particularly appropriate for determining utility values for rare diseases such as B-precursor ALL, where generating data from clinical trials can be challenging due to trial sample size or patient fitness and questionnaire burden. Of the techniques that can be used for preference elicitation, time trade-off (TTO) is the preferred method according to the guidelines of the National Institute for Health and Care Excellence (NICE) in the UK [8].

Considering the small patient population for B-precursor ALL and the lack of HRQL data in the literature, a utility study was designed in line with acceptable evidence in the NICE hierarchy. The purpose of this study was to define and validate health states experienced by adults with relapsed or refractory B-precursor ALL in the UK and to assign utility values to these health states, from the perspective of the UK general public, using the TTO methodology.

## Methods

### Study design

**Fig. 1:**
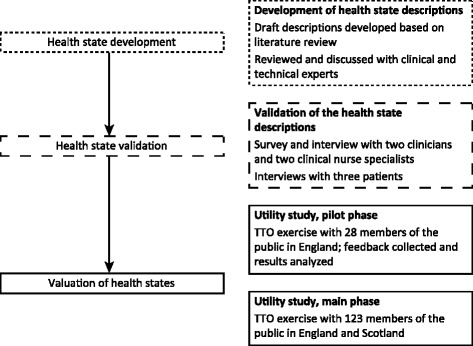
Overview of study procedures

This study was conducted in the UK between January 2013 and July 2014 (Fig. 1). The study was undertaken in three phases, described below: development of the health state descriptions, validation, and valuation. Valuation itself consisted of a two-part process: a pilot and a main preference elicitation study; the results from the main elicitation study only are presented here. The study design and all materials were reviewed and approved by the UK National Health Service Research Ethics Committee (reference 13/NW/0683).

### Development of the health state descriptions

Five health state descriptions were developed to describe the burden of B-precursor ALL and response to treatment: complete remission (CR), complete remission with partial hematological recovery (with minimal risk of bleeding or developing infection: platelets > 50,000/μl, and absolute neutrophil count (ANC) > 500/μl) (CRh), aplastic bone marrow (aBM), partial remission (PR), and progressive disease (PD). Development of health state descriptions was initially based on definitions from a single-arm Phase 2 clinical trial in 189 patients with relapsed or refractory B-precursor ALL, which defined all of the above as potential treatment outcomes and defined attainment of CR or CRh as the primary endpoint, (NCT01466179; [10]) and a literature review, which confirmed that there is no comprehensive HRQL information available for this patient population.

Each health state description included elements of symptoms and aspects of physical, functional, emotional, and social well-being, in line with the domains used in the FACT-Leu HRQL instrument [11]. The range for severity of symptoms depended on the health state being described. The structure and length of the descriptions were compared against health state descriptions published elsewhere in other cancers, such as chronic lymphocytic leukemia (CLL) [6], advanced melanoma [12], metastatic breast cancer [13], and chronic myeloid leukemia (CML) [14]. Adverse events that patients may experience during treatment were not included in the health state descriptions in order to keep the health state utility values independent of the treatment received. Terminology around cancer or leukemia was not included in the health state descriptions based on the conclusions of a study which found that the inclusion of a cancer label in health state descriptions negatively affects health state values [15].

A clinician experienced in the treatment of B-precursor ALL was then interviewed about the proposed health state definitions and based on this interview, the descriptions were amended to include the effect of hospital visits and the impact on a patient’s social life.

### Validation of the health state descriptions

To ensure their clinical accuracy and completeness, the five health state descriptions were next validated by two consultant hematologists and two clinical nurse specialists who had treated patients with relapsed or refractory B-precursor ALL in the previous 36 months, and three patients receiving treatment for B-precursor ALL.

The clinicians and nurses were provided with the health state descriptions and asked to give written feedback via a web-based structured questionnaire on whether the signs or symptoms of adult B-precursor ALL were correctly captured and whether the five health states descriptions based on treatment response were accurate. Comments from the health professionals were reviewed and summarized; majority decisions were taken for any contradictory comments between the health professionals. Based on the feedback, minor amendments were made to the descriptions; the most important aspects added included the patient’s typical emotional state and the frequency of hospital visits. It was concluded that including additional emotional aspects of the disease would aid comprehension of the health states, as some of the physical descriptions of the disease were relatively abstract (e.g., blood counts). Individual phone interviews were conducted with all the reviewers to confirm that proposed changes were appropriate.

The amended health state descriptions were then validated by three patients with B-precursor ALL. The patients were interviewed individually and face-to-face, using a semi-structured questionnaire, by an experienced NHS-based qualitative researcher with extensive experience of interviewing patients. The patients were asked to comment on the comprehensibility and face validity of the health state descriptions. The researcher asked the patients specifically about the impact of B-precursor ALL on their lives, the symptoms they experienced, and how these affected their personal and social lives. The patients also provided feedback on how clear the health state descriptions were, whether they reflected experiences patients may have, and whether any aspect was missing. The discussions were recorded and transcribed, and the transcripts analyzed by researchers to identify any consensus on the completeness and the comprehensiveness of the health state descriptions, and any supplementary descriptive items needed. Overall, the patients agreed with the health state descriptions, including the emotional aspects, and only minor amendments were made.

### Valuation of the health states

Health states were valued using a pilot and a main preference elicitation study, using TTO methods, with members of the general public in the UK.

Participants were recruited by a third-party vendor specializing in research with members of the public. Participants were recruited from London for the pilot study, and from London, Newcastle, and Edinburgh for the main study. Potential participants were screened based on age, sex, ethnicity, and socio-economic status to achieve a representative sample of the general UK population based on data from the Office of National Statistics 2011 Census for England and Wales [16].

Participants had to be at least 18 years of age at the time of the interview, able to understand the study procedures as judged by the investigator, and able to write and speak English. All participants were required to attend the interview in person. Exclusion criteria were severe cognitive impairment, hearing difficulty, visual impairment, or psychopathology as judged by the investigator. All participants provided informed consent and received compensation (fair market value) for their time and travel.

A pilot study was conducted with a small but representative sample of the general population in the UK (*n* = 28). The main aims were to confirm the utility elicitation methodology, ensure the health state descriptions were clear and easy to understand, and ensure participants are able to complete the interview in the time allotted.

Based on analysis of the pilot results, the following changes to the TTO exercise were made for the main part of the preference elicitation study:

- The health states underwent minor revisions to reduce the length and make differences between the states more clear.
- A ‘prior to treatment’ statement was designed to introduce the participants to what it would be like to have B-precursor ALL; this description was the same for each post-treatment health state. The amended health state descriptions were validated with one consultant hematologist and one specialist nurse prior to the main part of the preference elicitation study.
- Small simplifications to questionnaires were made; participants would be able to indicate their preference for a health state by circling it, whereas in the pilot study participants used ticks and crosses to indicate a preference as suggested in the MVH protocol (MVH Group, [20]).

The final health states used in the main part of the preference elicitation study are presented in Additional file 1.

In the main part of the preference elicitation study, participants completed the TTO questionnaires in group sessions comprising 15–17 individuals (8 sessions). Thus, four different versions of the questionnaire were generated, each containing all five of the health state descriptions, but presented in a unique order. Randomization was done by a statistical algorithm. During the exercise, health states were referred to as A, B, C, D and E, so as not to influence participant responses (see Additional file 1).

Participants in each session were provided with written and verbal descriptions of the exercise, and instructions on how to complete the TTO questionnaire, which was demonstrated with an example. After completing standard demographic questions, participants were asked to rate their own health on the day using the EQ-5D. The participants then rated the health state descriptions for relapsed/refractory B-precursor ALL.

### Preference elicitation

The valuation exercise in both the pilot and main study was accomplished through self-completion of a paper-based TTO questionnaire in a moderated group session, based on the MVH protocol modelling of valuation tariffs [17] (see Additional file 2). Trained moderators were available to answer participants’ questions throughout the exercise.

For each health state, participants were asked to state whether they would prefer to live in the health state described for 10 years, followed by death, or whether they would trade any of the 10 years to live a shorter time in full health. Participants completed a table in which they could choose between “perfect health” or a particular health state, where the health state lasted for 10 years and the duration of perfect health was a progressively shorter period in each row, ending at zero (i.e., death). The point at which the participant could not choose between perfect health and the health state was considered the point of indifference and used to calculate the utility score. If participants did not reach this point of indifference, the first time a participant choose perfect health was used in the calculations for the utility score.

In accordance with the MVH protocol, if participants preferred immediate death to living in a health state for 10 years, they were asked to complete another table in which they were asked to choose between immediate death and combinations of time in the health state followed by perfect health (for a combined duration of 10 years). Increasing periods of time in the health state (with decreasing periods of time in perfect health) were presented to find the point of indifference among these choices.

Although patients with relapsed or refractory B-precursor ALL generally have a much shorter life span, the 10-year time trade-off was used to align with the MVH York protocol.

### Statistical analysis

Quantitative analysis that generated descriptive statistics (mean, standard deviation, and frequency) was used to summarize the utility values and characterize the sample in terms of socio-demographic and other characteristics. The study responses were also analyzed descriptively (mean and standard deviation) to determine average time to completion and the difference in value between health states. The study was not powered to test for statistically significant differences between subgroups or between the study population and previously published data from the UK general population.

The health states were scored as follows:

- For states better than death, the numbers of years of perfect health that respondents would trade for 10 years in each health state were converted to a relative utility value using a simple fraction: (10 – t)/10, where t was the amount of time in years that was traded [17].
- For states worse than death, scores were converted to a relative utility value using the transformation: t’ = − t/(10–t), where t is the amount of time spent in full health [17]. These values were then truncated at −1, which replaced all values below −1. The mean utility was calculated using the truncated values.

## Results

In total, 123 participants (55 from London, 25 from Edinburgh, and 43 from Newcastle) were recruited to the main study and included in the final analysis. The demographic profile of the study population was similar to the 2011 census data for sex, age, ethnicity, economic activity, and marital status (Table 1). The household size, household income, and education level of the study population were slightly higher than those of the UK general population. Tests for statistically significant differences in demographics between centers, or between this study and the UK general population, were not conducted.

**Table 1:** Sex, age, ethnicity, and economic activity of the study sample (*n* = 123)

	Participants, n (%)	Census 2011 (%)
Sex		
M:F	62 (50.4):61 (49.4)	49: 51
Age group (years)		
18–29	23 (18.7)	20.6
30–44	34 (27.6)	26.1
45–59	34 (27.6)	24.8
60–74	27 (22.0)	18.7
75+	5 (4.1)	9.8
Ethnicity		
White (British or other white)	108 (87.8)	87.1
Non-white	15 (12.2)	12.9
Economic activity		
Full-time work (30+ hours)	60 (48.8)	37.4
Part-time work	11 (8.9)	12.6
Self-employed	8 (6.5)	8.5
Full-time education	7 (5.7)	3.5
Retired	22 (17.9)	22.1
Looking after home	5 (4.1)	3.3
Long-term illness/disabled	2 (1.6)	3.4
Unemployed/seeking work	4 (3.3)	8.3
Other	4 (3.2)	1.2
Marital status		
Single	42 (34.1)	34.7
Married/Civil partnership	56 (45.5)	46.7
Divorced/Separated/Legally dissolved	22 (17.9)	11.5
Widowed	3 (2.4)	7.0
Household size		
1	27 (22.0)	30.6
2	32 (26.0)	34.1
3–4	24 (39.0)	28.5
5–6	16 (13.0)	6.2
Household income (£)		
0–19,999	26 (23.6)	49.9
20,000–29.999	19 (17.3)	22.7
30,000–49,999	42 (38.2)	18.6
50,000–99,999	20 (18.2)	6.7
100,000 or more	3 (2.7)	2.2
Preferred not to answer	13 (10.5)	NA
Education level		
No formal qualifications	11 (8.9)	23.2
Apprenticeships	6 (4.9)	3.3
High school education	72 (58.5)	41.4
University degree or professional qualification	31 (25.2)	27.0
Other	3 (2.4)	5.1

Source: [16]

The completion rate for the questionnaire was 100 %; participants took an average of 25 min (range: 9–53 min) to complete the questionnaire. All participants indicated that they understood the health state descriptions and were able to score them using the TTO questionnaire, although one participant noted that it was difficult to imagine the cumulative impact of the symptoms described.

The mean EQ-5D score for participants was 0.91 (SD 0.17), which is slightly higher than previously published EQ-5D scores for the UK general population: 0.86 (SD 0.23; [18] and 0.868 (range:−0.594 to 1; [19]). Tests for statistically significant differences in EQ-5D scores between this study and the UK general population were not conducted.

Complete remission and complete remission with partial hematological recovery (with minimal risk of bleeding or developing infection) were associated with the highest mean utility values (SEM): 0.86 (0.01) and 0.75 (0.02), respectively, indicating the lowest HRQL burden (Table 2). The progressive disease state had the lowest values (0.30 [0.04]), indicating the highest burden. Aplastic bone marrow and partial remission had mean values of 0.59 (0.02) and 0.50 (0.03), respectively.

**Table 2:** Mean TTO utility values for disease-related health states in B-precursor ALL (*n* = 123)

Health state	Mean TTO utility value^a^	TTO SEM	Increment^b^	Increment SEM
Complete remission	0.86	0.01	0.56	0.042
Complete remission with partial hematological recovery	0.75	0.02	0.45	0.038
Aplastic bone marrow	0.59	0.02	0.29	0.034
Partial remission	0.50	0.03	0.20	0.032
Progressive disease	0.30	0.04	–	–

*Abbreviations*: *SEM* standard error of the mean, *TTO* time trade-off

^a^Health state utility values range from 0 to 1; 0 is death and 1 is perfect health

^b^Progressive disease was the anchoring health state for calculation of increments

Nine participants scored progressive disease as worse than death; two participants valued partial remission as worse than death; and one participant considered aplastic bone marrow to be worse than death.

## Discussion

To our knowledge, this is the first study to generate utility values for health states related to adult relapsed or refractory B-precursor ALL. As expected, preferences for treatment response states (i.e., complete remission or complete remission with partial hematological recovery) were substantially higher than for progressive disease, and decreased logically with increasing disease severity. Previous utility studies of advanced or rare cancers have generally demonstrated that health states for treatment response and stable disease are preferred to progressive disease [6, 12, 14], as these states are associated with a lower burden on patients. These results indicate that the general public were able to differentiate and value clinical health states for patients with relapsed or refractory B-precursor ALL. The utilities generated can therefore be used in future cost-effective evaluations of treatments for relapsed or refractory B-precursor ALL in adults.

The study implemented methodology to align with NICE guidance on alternative methods for generating health state utility values when EQ-5D data are either unavailable or inappropriate. After confirming the lack of utility values for adult relapsed or refractory B-precursor ALL through a systematic search of the literature, we developed and validated five health states for the disease. The validation process incorporated interviews with consultant hematologists, clinical nurses and three patients with B-precursor ALL, despite the rarity of the disease. The descriptions therefore captured important elements of the patient experience, ensuring strong content and face validity. Finally, we used TTO during the valuation phase and followed standard procedures for the exercise and the analysis of the results [17, 20]. Notably, utility values for other types of leukemia have been elicited using similar methodology [21, 22]. Although these studies were in chronic leukemia (CLL and CML), their use of preference elicitation highlights the lack of HRQL data for leukemia and supports our approach generally. Overall, this methodology meets the standards set out in NICE guidance, and is a key strength of the study.

NICE have stated that health state utility values for participants should be comparable with values from the UK general population, to ensure the face validity of the health state valuations [7, 8]. Although the utility value for complete remission in this study was the same as reported for the average of the UK general population (0.86), it should be noted that the self-rated utility of the study sample was higher (0.91). A slightly healthier sample is generally expected in this type of study, where participants are required to attend a study site and the rank order of the study health states are logical. The health state descriptors do not take into account comorbidities present in the UK general population, which would potentially reduce valuations. Further, the health state descriptions must be relatively short for this type of study, and may therefore have excluded elements that could potentially have influenced valuations.

Utility values below −1 were truncated in the base case analysis, but several alternative methods for handling these values exist and the best method is subject to debate [23–26]. In theory, there is no lower bound on the utility values for states worse than death. Lamers and colleagues consider that median values should be used, but NICE guidance states that health state valuation for economic evaluation to inform decision-making has been based mainly on mean values to date [25, 27]. Mean values give all individuals a weighting that is proportional to them, which is important in the valuation of health states because the values should reflect the preferences of the whole population, and not consider some valuations to be more accurate than others.

The sample was generally representative of the UK population. A higher proportion of respondents had annual incomes of £50,000–99,999 than in the 2011 census (18.7 versus 6.7 %) and a lower proportion had no formal qualifications (8.9 versus 23.2 %). This may be related to the greater number of participants from London (*n* = 55), where living costs are high. However, these demographic variables have not been shown to be reliable predictors of health state utilities and are not expected to have had a significant effect on the valuation of the health state descriptions [17]. Importantly, the age distribution in the study sample closely matches that of the UK population, with the exception of elderly participants, who are slightly underrepresented. This is perhaps due to the face-to-face methodology; elderly people may have had more difficulty in attending the study site. Age has been shown to influence valuations of health [17]; adults over 60 years old tend to regard many health states as being much worse than the rest of the population. The MVH Group found that some of this difference may be due to older people believing they would never regain full health after severe disease states; however some of the difference was also genuine. As such, including a higher proportion of elderly people may have slightly lowered our utility values.

## Conclusions

This large study used a methodology in line with NICE guidance to elicit utility values for health states in relapsed or refractory adult B-precursor ALL from the UK general population. The utility values generated can be applied in cost–utility analyses of treatments for this condition.

## Additional files

Additional file 1:
**Health state descriptions.** (DOCX 41 kb) Additional file 2:
**Survey instrument: time trade-off exercise.** (DOCX 58 kb)
