# Supplementary material for: Population preference values for health states in relapsed or refractory B-precursor acute lymphoblastic leukemia in the United Kingdom
Source: Health Qual Life Outcomes. 2015 Nov 16;13:181. doi: 10.1186/s12955-015-0377-3 (PMC4647711; doi:10.1186/s12955-015-0377-3)
Supplement: Additional file 1: — Health state descriptions. (DOCX 41 kb) [file 12955_2015_377_MOESM1_ESM.docx]

# Appendix 2. Health State Descriptions

Five health state descriptions were developed to describe the burden of B-precursor ALL and response to treatment: complete remission (CR); complete remission with partial hematological recovery (CRh); blast-free hypoplastic or aplastic bone marrow (aBM); partial remission (PR); progressive disease (PD).

## Complete remission (Health state C)

| Prior to treatment | Upon achieving a complete response |
| --- | --- |
| You have a serious illness that is life-threatening and affects your wellbeing. This disease weakened your immune system and its ability to fight infection, thereby increasing the risk of you developing serious infections which would require hospitalization and could lead to death. Additionally, you are constantly experiencing severe, debilitating symptoms that are keeping you from living the life you were prior to developing the disease. These severe symptoms include some or all of the following:   - You feel tired or weak - Your appetite is affected - You are bothered by sudden fevers (episodes of high body temperature) - You have trouble sleeping - You feel short of breath during normal activities - You have night sweats - You have swollen glands in your neck, armpits, or groin - Your joints and bones hurt - You may have unexplained bruises - You are not able to perform normal daily activities | You have had a serious illness that was life-threatening and affected your well-being. The disease weakened your immune system, thereby increasing the risk of you developing serious infections, which would require hospitalization and could lead to death   - Your treatment has worked, and your doctor considers that you no longer have signs of the disease - You may still experience some symptoms such as fatigue - Your doctor has explained that your blood tests have returned to normal and hence you are no longer at risk for developing serious infections - You feel relieved because your treatment has worked - You have a good chance of going back to work in the near future - You are socializing normally again (e.g., meeting friends and family, going to the cinema or restaurants) |

## Complete remission with partial hematological recovery (Health state D)

| Prior to treatment | Upon achieving a CRh |
| --- | --- |
| You have a serious illness that is life-threatening and affects your wellbeing. This disease weakened your immune system and its ability to fight infection, thereby increasing the risk of you developing serious infections which would require hospitalization and could lead to death. Additionally, you are constantly experiencing severe, debilitating symptoms that are keeping you from living the life you were prior to developing the disease. These severe symptoms include some or all of the following:   - You feel tired or weak - Your appetite is affected - You are bothered by sudden fevers (episodes of high body temperature) - You have trouble sleeping - You feel short of breath during normal activities - You have night sweats - You have swollen glands in your neck, armpits, or groin - Your joints and bones hurt - You may have unexplained bruises - You are not able to perform normal daily activities | You have had a serious illness that was life-threatening and affected your well-being. The disease weakened your immune system, thereby increasing the risk of you developing serious infections, which would require hospitalization and could lead to death   - Your treatment has worked, and your doctor considers that you no longer have signs of the disease, but some of your blood counts are below the normal range - You still have some residual symptoms such as fatigue - While your risk of infection has decreased, there is still a chance of developing a serious infection - You feel relieved because your treatment has worked - You have a chance of going back to work in the near future. - You are cautious about socializing (e.g., meeting friends and family, going to the cinema or restaurants) because you don’t want to catch a cold or flu. |

Blast-free hypoplastic or aplastic bone marrow (Health state A)

| Prior to treatment | Upon achieving blast-free hypoplastic or aplastic bone marrow |
| --- | --- |
| You have a serious illness that is life-threatening and affects your wellbeing. This disease weakened your immune system and its ability to fight infection, thereby increasing the risk of you developing serious infections which would require hospitalization and could lead to death. Additionally, you are constantly experiencing severe, debilitating symptoms that are keeping you from living the life you were prior to developing the disease. These severe symptoms include some or all of the following:   - You feel tired or weak - Your appetite is affected - You are bothered by sudden fevers (episodes of high body temperature) - You have trouble sleeping - You feel short of breath during normal activities - You have night sweats - You have swollen glands in your neck, armpits, or groin - Your joints and bones hurt - You may have unexplained bruises - You are not able to perform normal daily activities | You have had a serious illness that was life-threatening and affected your well-being. The disease weakened your immune system, thereby increasing the risk of you developing serious infections, which would require hospitalization and could lead to death   - Your treatment has worked and you are in remission, but your blood counts are below the normal range and your body is not making new blood cells appropriately - You still have some symptoms, and some may be severe such as bleeding and you may need regular blood transfusions - You may still need antibiotics to reduce the risk of developing a serious infection - You sometimes worry about dying and you may feel depressed - You are not sure whether you will be able to go to work in the future - You are cautious about socializing (e.g., meeting friends and family, going to the cinema or restaurants) because you don’t want to catch a cold or flu |

Partial remission (Health state E)

| Prior to treatment | Upon achieving a partial response |
| --- | --- |
| You have a serious illness that is life-threatening and affects your wellbeing. This disease weakened your immune system and its ability to fight infection, thereby increasing the risk of you developing serious infections which would require hospitalization and could lead to death. Additionally, you are constantly experiencing severe, debilitating symptoms that are keeping you from living the life you were prior to developing the disease. These severe symptoms include some or all of the following:   - You feel tired or weak - Your appetite is affected - You are bothered by sudden fevers (episodes of high body temperature) - You have trouble sleeping - You feel short of breath during normal activities - You have night sweats - You have swollen glands in your neck, armpits, or groin - Your joints and bones hurt - You may have unexplained bruises - You are not able to perform normal daily activities | You have a serious illness that is life-threatening and is affecting your well-being. The disease weakened your immune system, thereby increasing the risk of you developing serious infections, which would require hospitalization and could lead to death.   - Your treatment has reduced your signs and symptoms of disease, but your doctor has explained that you still have the disease. - You still have a chance of developing a serious infection - You still have most symptoms and you know this illness will get worse - You receive treatments on a daily basis in a hospital or an outpatient clinic - You sometimes worry about dying and you feel quite depressed - You are unlikely to be able to go back to work in the future |

Progressive disease (Health state B)

| Prior to treatment | Upon progressive disease |
| --- | --- |
| You have a serious illness that is life-threatening and affects your wellbeing. This disease weakened your immune system and its ability to fight infection, thereby increasing the risk of you developing serious infections which would require hospitalization and could lead to death. Additionally, you are constantly experiencing severe, debilitating symptoms that are keeping you from living the life you were prior to developing the disease. These severe symptoms include some or all of the following:   - You feel tired or weak - Your appetite is affected - You are bothered by sudden fevers (episodes of high body temperature) - You have trouble sleeping - You feel short of breath during normal activities - You have night sweats - You have swollen glands in your neck, armpits, or groin - Your joints and bones hurt - You may have unexplained bruises - You are not able to perform normal daily activities | You have a serious illness that is life-threatening and is affecting your well-being. The disease weakened your immune system, thereby increasing the risk of you developing serious infections, which would require hospitalization and could lead to death.   - Your treatment has not worked and your disease is getting worse. You are not likely to receive curative treatment - Your signs and symptoms are getting worse - You are aware you are at risk of serious infections and these could lead to blood poisoning (sepsis), which is life-threatening - You receive supportive treatment to relieve some of your symptoms - You often worry about dying and you feel very depressed - You are worried about how your death will affect the people around you |
