# Supplementary material for: Population preference values for health states in relapsed or refractory B-precursor acute lymphoblastic leukemia in the United Kingdom
Source: Health Qual Life Outcomes. 2015 Nov 16;13:181. doi: 10.1186/s12955-015-0377-3 (PMC4647711; doi:10.1186/s12955-015-0377-3)
Supplement: Additional file 2: — Survey instrument: time trade-off exercise. (DOCX 58 kb) [file 12955_2015_377_MOESM2_ESM.docx]

# Appendix 2. Survey instrument: time trade-off exercise

Time Trade Off: Health State X

1. Now we would like to explore how good or bad health state X is using a Time Trade-Off exercise. Think of yourself in health state X. Imagine that you expect to live for another 10 years. Also imagine that you could be given a cure, which will restore you to perfect health. BUT the cure will shorten your life expectancy. This means that you will not live the remaining 10 years of your life, but, during the remainder of your shorter life you will live in perfect health. The table below indicates the life years you would have in perfect health or health state X. For each row, we would like you to indicate which option you prefer.

For each row, we would like you to indicate which option you prefer by circling your choice i.e. perfect health for reduced years (left column) or health state D for 10 years (middle column). Where you cannot choose between the options in a row, please place an equal sign ‘=’ (right column).

| Perfect health |  | Health state X | Where cannot choose ‘=’ |
| --- | --- | --- | --- |
| 10 years | or | 10 years |  |
| 9 years 6 months | or | 10 years |  |
| 9 years | or | 10 years |  |
| 8 years 6 months | or | 10 years |  |
| 8 years | or | 10 years |  |
| 7 years 6 months | or | 10 years |  |
| 7 years | or | 10 years |  |
| 6 years 6 months | or | 10 years | = |
| 6 years | or | 10 years |  |
| 5 year 6 months | or | 10 years |  |
| 5 years | or | 10 years |  |
| 4 years 6 months | or | 10 years |  |
| 4 years | or | 10 years |  |
| 3 years 6 months | or | 10 years |  |
| 3 years | or | 10 years |  |
| 2 years 6 months | or | 10 years |  |
| 2 years | or | 10 years |  |
| 1 years 6 months | or | 10 years |  |
| 1 years | or | 10 years |  |
| 0 years 6 months | or | 10 years |  |
| 0 years | or | 10 years |  |

Please now consider the bottom row of the table.

- If you have answered an ‘=’ in the right column on the bottom row answer question 2
- If you have circled ‘0 years’ in left column on the bottom row, this suggests you think health state D is worse than immediate death. Skip to question 3
- If you have circled ‘10 years’ in the middle column of the bottom row, skip to the next health state

2. Do you think health state D is worse than immediate death?

No; please go to the next health state Yes; please answer question 3

3. Suppose you were to live instead with health state D for a period of time, followed by perfect health, for a total of 10 years as indicated in the table below. For each row, please indicate which option you prefer.

For each row, we would like you to indicate which option you prefer by circling your choice i.e. health state D followed by perfect health perfect health for reduced years (left columns) or immediate death (middle column). Where you cannot choose between the options in a row, please place an equal sign ‘=’ (right column).

| Health state B | Perfect health | Answer | Immediate death |
| --- | --- | --- | --- |
| 0 years 6 months | 9 years 6 months |  | Immediate death |
| 1 year | 9 years |  | Immediate death |
| 1 years 6 months | 8 years 6 months |  | Immediate death |
| 2 years | 8 years |  | Immediate death |
| 2 years 6 months | 7 years 6 months |  | Immediate death |
| 3 years | 7 years |  | Immediate death |
| 3 years 6 months | 6 years 6 months |  | Immediate death |
| 4 years | 6 years |  | Immediate death |
| 4 years 6 months | 5 years 6 months |  | Immediate death |
| 5 years | 5 years |  | Immediate death |
| 5 years 6 months | 4 years 6 months |  | Immediate death |
| 6 years | 4 years |  | Immediate death |
| 6 years 6 months | 3 years 6 months |  | Immediate death |
| 7 years | 3 years |  | Immediate death |
| 7 years 6 months | 2 years 6 months |  | Immediate death |
| 8 years | 2 years |  | Immediate death |
| 8 year 6 months | 1 years 6 months |  | Immediate death |
| 9 years | 1 year |  | Immediate death |
| 9 year 6 months | 0 years 6 months |  | Immediate death |
| 10 years | 0 years |  | Immediate death |
